# Suppressive Effects of Isofraxidin on Depressive-like Behaviors Induced by Chronic Unpredictable Mild Stress in Mice

**DOI:** 10.3390/brainsci12101376

**Published:** 2022-10-11

**Authors:** Xiaoli Wu, Jingwen Gu, Zhicong Zou, Meng Yu, Chen Zhang, Qinghui Xiao, Xin Chen, Chuwen Li

**Affiliations:** 1 School of Biomedical and Pharmaceutical Sciences, Guangdong University of Technology, Guangzhou 510006, China; 2 School of Pharmaceutical Sciences, Guangzhou Medical University, Guangzhou 511436, China; 3 School of Automation, Guangdong University of Technology, Guangzhou 510006, China

**Keywords:** isofraxidin, depression, CUMS, HPA axis, neuroinflammation, NF-κB-NLRP3 inflammasomes

## Abstract

Isofraxidin is an active component of several traditional and functional plants that have beneficial properties for neurodegenerative diseases. In this study, we examined whether isofraxidin exhibited antidepressant-like effects in chronic unpredictable mild stress (CUMS)-induced mice. Firstly, isofraxidin could reverse CUMS-induced decrease in body weight gain in mice. Additionally, in the sucrose preference test (SPT), isofraxidin reversed the decrease in sucrose consumption due to CUMS-induced depressive-like behavior. Isofraxidin also increased locomotor activity in the open field test (OFT) and alleviated immobility duration in the forced swimming test (FST) and tail-suspension test (TST). Furthermore, isofraxidin decreased levels of corticosterone (CORT), adrenocorticotropic hormone (ACTH), and hypothalamus corticotrophin-releasing hormone (CRH) in the serum after CUMS-induced hyperactivity of the hypothalamic-pituitary-adrenal (HPA) axis. Also, isofraxidin suppresses tumor necrosis factor (TNF)-α, interleukin (IL)-1β, and IL-6 expression in the hippocampus of CUMS mice. Further investigations demonstrated that isofraxidin inhibited CUMS-induced activation of nuclear factor kappa B (NF-κB) and NOD-like receptor protein 3 (NLRP3) inflammasomes in the hippocampus. Summarily, in CUMS-induced mice, isofraxidin reduced depressive-like behaviors, accompanied by its inhibitory effects on hyperactivity of the HPA axis and NF-κB /NLRP3 inflammasomes pathways.

## 1. Introduction

Major depressive disorder, often characterized as depression, is a prevalent mental condition [1,2]. The high rates of illness, recurrence, and suicide associated with depression represent a serious risk to the public’s health. According to the World Health Organization, depression is a common illness worldwide, with an estimated 3.8% of the population affected, including 5.0% among adults and 5.7% among adults older than 60 years [1,2]. Despite the progression of depression and its underlying causes, currently available antidepressants have glaring drawbacks, such as limited effectiveness, medication resistance, and significant adverse effects. Therefore, the creation of innovative medicines for the treatment of depression remains essential.

It is commonly known that stress can affect the production of numerous critical components of the hypothalamic-pituitary-adrenal (HPA) axis, such as corticosterone (CORT), adrenocorticotropic-hormone (ACTH), and corticotrophin-releasing-hormone (CRH) [3,4]. The hyperactivity of the HPA axis is caused by the excess of these hormones, which sets off a negative cycle that causes the HPA axis to express additional hormones [5,6]. Accordingly, depressive disorders are invariably accompanied by hyperactivity in the HPA axis in both clinical investigations and animal models [5,6]. As a result of disruption in the HPA axis, fewer neurotransmitters and neuroprotective factors are secreted, which eventually involves depression [3,4,5,6]. Moreover, inhibition of HPA axis hyperactivity represents an important mechanism of several currently-used antidepressants [7,8]. Consequently, manipulation of the HPA axis indicates a potential depression therapeutic target.

A growing body of studies demonstrates that neuroinflammation has a role in the development of depression [9,10]. According to several pieces of clinical research, the hippocampus and cerebrospinal fluid of depressed individuals and animals both have considerably higher levels of inflammatory cytokines, including tumor necrosis factor (TNF)-α, interleukin (IL)-1β, and IL-6 [9,10]. These excessive pro-inflammatory cytokines have been linked to cellular harm, impairment of brain plasticity, and inhibition of neurogenesis [9,10]. Nuclear factor kappa B (NF-κB) and NOD-like receptor protein 3 (NLRP3) inflammasomes are the regulators of several inflammatory processes, which participate in the control of cytokines and mediators [11,12,13,14,15]. Once NF-κB is activated, it induces transcription and expression of pro-inflammatory cytokines [13,14,15]. The NLR family of NLRP3 inflammasomes is mainly composed of NLRP3 and apoptosis-associated speck-like protein containing (ASC) [16,17,18,19]. NLRP3 is a component of the innate immune system, and its primary function is to regulate the activation of caspase-1 and IL-1β [16,17,18,19]. Consequently, modulating NF-κB/NLRP3 pathways represents an important strategy for the development of antidepressants. 

**Figure 1:**
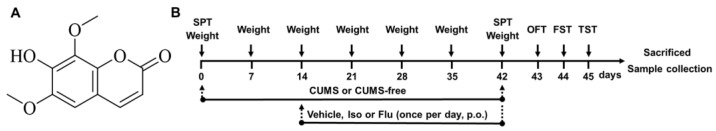
The chemical structure and experimental design. (**A**) The chemical structure of isofraxidin. (**B**) The experimental protocol. CUMS, chronic unpredictable mild stress. Iso, Isofraxidin. FLU, fluoxetine hydrochloride. SPT, sucrose preference test. OFT, open field test. FST, forced swimming test. TST, tail suspension test.

Isofraxidin (7-hydroxy-6,8-dimethoxycoumarin, the chemical structure is shown in Figure 1A) is mostly found in traditional herbs and functional foods, such as *Sarcandra glabra*, *Apium graveolens*, and *Siberian Ginseng* [20]. These herbs have been extensively used in traditional herbal therapy to treat various kinds of central nervous system diseases [20]. Our previous study indicates isofraxidin can alleviate cognitive and memory impairments in mice with dementia [21]. Accumulating evidence also demonstrates that it exerts significant anti-inflammatory activities in several inflammatory models via regulating NF-κB and NLRP3-related pathways [22,23,24]. Additionally, isofraxidin possesses considerable anti-oxidative activity models in vitro and in vivo [25,26]. The effects of isofraxidin on neuroinflammation and oxidative stress suggest that it can prevent and cure depressive-like conditions. Chronic unpredictable mild stress (CUMS)-induced depressive models are extensively utilized for testing antidepressant effectiveness [27,28,29,30,31,32,33]. In this work, we sought to examine the effects of isofraxidin on depression in mice induced by CUMS. For the examination of putative mechanisms, NF-κB/NLRP3 inflammasomes pathways were also investigated.

## 2. Materials and Methods

### 2.1. Materials Chemicals and Reagents

Isofraxidin (Iso, power, purity: ≥98%) was purchased from Weikeqi (Sichuan, China). Fluoxetine hydrochloride (Flu, power, purity: ≥98%) was obtained from J&K Scientific (Beijing, China). ELISA Kits were provided by MyBioSource (San Diego, CA, USA). Primary antibodies such as NF-κB p65, NLRP3, ASC, caspase-1, β-actin, and GAPDH were obtained from Cell Signaling Technology (Beverly, MA, USA).

### 2.2. Animals and Treatments

Male BALB/c mice (about 8 weeks old, 18–20 g) were obtained from the Medical Laboratory Animal Center of Guangdong Province (Certificate number SCXK2008-0002, Guangdong Province, China). The animals were housed at a temperature (20 ± 2 °C), humidity (50 ± 10 %), and light/dark cycle (12-h light/12-h dark), and were provided with a portion of regular food and clean water. All protocols for animal experiments were conducted under institutional ethical approval of the Experimental Animal Center, Guangzhou Medical University (GY2020-055).

The mice were then divided into 7 groups (10 mice per group), including control (normal saline), Iso (30 mg/kg of isofraxidin), CUMS (normal saline), CUMS, Iso (3, 10, and 30 mg/kg of isofraxidin), and CUMS and Flu (10 mg/kg of fluoxetine hydrochloride). Flu is a classical antidepressant and is used for the treatment of the major depressive disorder. Flu has been widely used as a positive drug in various kinds of depressive animal models. The CUMS-free animals were kept in a separate room, with no contact with the CUMS mice. The other mice suffered from CUMS for a period of 6 weeks. Isofraxidin or fluoxetine hydrochloride was daily administered by oral gavage from the second week, lasting for 4 weeks. Details are illustrated in Figure 1.

### 2.3. CUMS

The CUMS process was executed with minor adjustments according to previous publications [30,31,32,33]. The CUMS groups were exposed to CUMS every day from 11:00 a.m. to 12:00 p.m. To make the procedure unpredictable, procedures were scheduled arbitrarily and altered weekly. The stressors include (a) cage tilting (45°) for 12 h, (b) swimming in cold water (4 °C) for 5 min, (3) food deprivation for 12 h, (4) water deprivation for 12 h, (c) white noise for 12 h, (d) forced physical restraint for 5 min, (e) damp bedding for 12 h, (f) foreign objects for 12 h, and (g) reversed light/dark cycle (illumination overnight).

### 2.4. Behavioral Tests

#### 2.4.1. Sucrose Preference Test (SPT)

The SPT was administered using protocols previously reported with slight modifications [30,31,32,33]. Animals were deprived of food and drink for 12 h ahead of the test. The animals were then given the option between two bottles, one containing a 2% (*w*/*v*) sucrose solution and the other containing water. The placements of these two bottles were rearranged every six hours to eliminate the possibility of beverage preferences. After 24 h, the consumption was assessed by weighing each bottle, and the total liquid intake and sucrose preference rate (SPR) were determined. The calculation formula of SPR = sucrose intake/(sucrose intake + water intake) × 100%.

#### 2.4.2. Open Field Test (OFT)

We assessed the actions of isofraxidin and CUMS on spontaneous locomotor activity using OFT, as described in previous reports [30,31,32,33]. The device was divided into 12 equal squares (500 × 500 × 500 mm). First, animals were put in the center for 5 min to acclimate to the surroundings. During these 5 min, spontaneous locomotor activity (immobility time, movement distance, and percentage of time passing the center) was recorded and measured. During each trial, the floor and walls were properly sanitized with ethanol solution and dried completely.

#### 2.4.3. Forced Swimming Test (FST)

The FST was carried out as previously reported with minor adjustments [30,31,32,33]. In brief, animals were put individually in a transparent glass cylinder (200 × 200 mm) containing water at about 25 °C. The immobility time was defined as the duration that mice floated in water without resisting or making a motion to maintain their heads above water. After adapting each mouse for 5 min, the cumulative length of immobility in the subsequent 5 min was recorded.

#### 2.4.4. Tail-Suspension Test (TST)

The TST was conducted as previously reported [30,31,32,33]. Animals were hung by their tails from an adhesive tape-lined ledge 50 cm above a tabletop for 5 min. During a 5-min test, each animal was acclimated for 1 min, and the remaining 4 min were used to record the duration of immobility. Animals were only immobile when hanging quietly and were entirely motionless.

### 2.5. Sample Collections

Following behavioral examinations, mice were then randomly divided into 2 portions (5 mice per portion). One portion was euthanized with isoflurane. Then, blood samples were obtained and serum separations were performed. Another portion was sacrificed by inhaling carbon dioxide. Then, hippocampus and hypothalamus were isolated, quickly frozen with liquid nitrogen, and kept at −80 °C.

### 2.6. Enzyme-Linked Immunosorbent Assay (ELISA)

Concentrations of CORT, ACTH, CRH, and hippocampal concentrations of TNF-α, IL-6, and IL-1β were evaluated using ELISA kits according to the instructions. Finally, the data were standardized based on total protein content.

### 2.7. Western Blot Analysis

The samples were lysed in RIPA lysis buffer, followed by centrifugation at 13,500 rpm at 4 °C for 20 min. The supernatants were obtained and the protein content was determined. Protein was separated by SDS-PAGE (7.5–15% resolution) and transferred to the PVDF membrane. The membrane was blocked by 5% BSA, then incubated overnight at 4 °C with diluted primary antibodies. Membranes were cleaned and exposed to the secondary antibody for one hour at room temperature. Finally, the bands were seen and analyzed utilizing a Bio-Rad ChemDoc XRS Imaging System (Bio-Rad, Hercules, CA, USA).

### 2.8. Statistical Analysis

GraphPad Prism software (V8.0, GraphPad, San Diego, CA, USA) was used to conduct statistical analyses, and data are provided as means standard error of the mean (SEM). The one-way ANOVA with Bonferroni correction was employed for data analysis. A *p <* 0.05 was deemed statistically significant.

## 3. Results

### 3.1. Isofraxidin Reverses CUMS-Induced Decrease in Body Weight Gain

**Figure 2:**
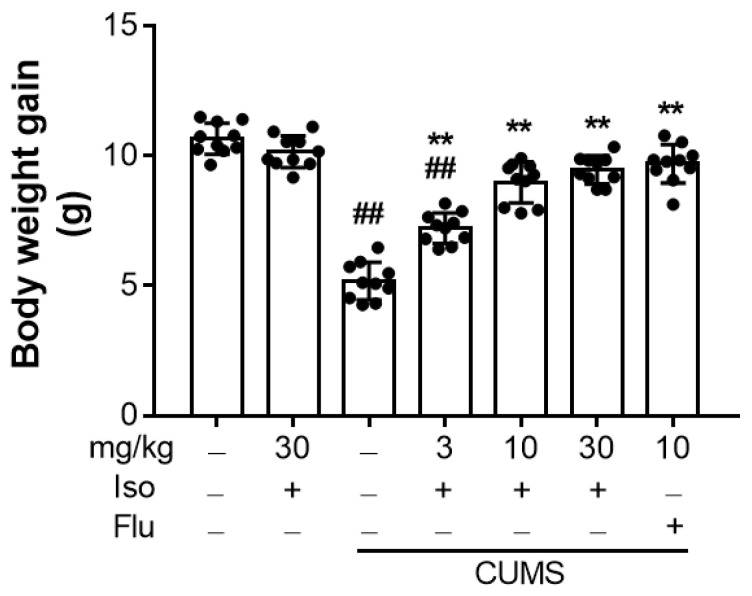
Isofraxidin reversed CUMS-induced decrease in body weight gain. Body weight gain of mice at Day 42. Data are presented as means ± SEM (*n* = 10). The Control group was CUMS-free mice administered with normal saline. CUMS group was CUMS mice. Significance was analyzed by one-way ANOVA followed by the Bonferroni correction. ## *p <* 0.01, compared with the control group. ** *p <* 0.01, compared with CUMS group.

As illustrated in Figure 2, the increase in body weight in the CUMS group was substantially lower than that in the control group (F (6, 63) = 84.12, *p <* 0.01). However, treatments with isofraxidin ((F (6, 63) = 84.12; for 3, 10 and 30 mg/kg, all *p <* 0.01) or fluoxetine hydrochloride ((F (6, 63) = 84.12; for 10 mg/kg, *p <* 0.01) significantly reversed the decrease in the body gain, compared with the CUMS group.

### 3.2. Isofraxidin Restores CUMS-Induced Decrease in Sucrose Consumption

**Figure 3:**
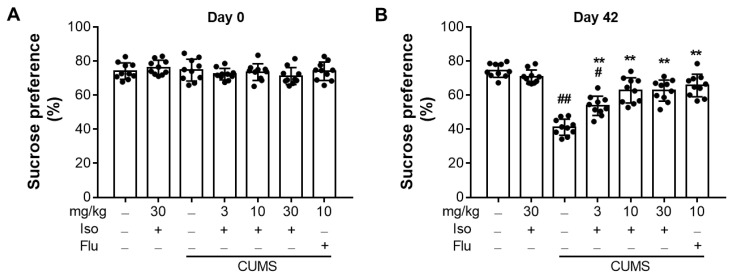
Isofraxidin restores CUMS-induced decrease in sucrose consumption. (**A**) Baseline sucrose consumption ratio at Day 0. (**B**) Sucrose consumption at Day 42. Data are presented as means ± SEM (*n* = 10). The Control group was CUMS-free mice administered with normal saline. CUMS group was CUMS mice. Significance was analyzed by one-way ANOVA followed by the Bonferroni correction. # *p <* 0.05 and ## *p <* 0.01, compared with the control group. ** *p <* 0.01, compared with CUMS group.

The SPT was administered before and 6 weeks following CUMS. As shown in Figure 3A, there was no significant difference in the baseline of SPT scores (F (6, 63) = 1.064; for day 0, all *p >* 0.05). After six weeks of CUMS, the CUMS group’s consumption was considerably lower than that of the control group (Figure 3B; F (6, 63) = 39.02; *p <* 0.01) However, treatment with isofraxidin (F (6, 63) = 39.02; for 3, 10 and 30 mg/kg, all *p <* 0.01) or fluoxetine hydrochloride (F (6, 63) = 39.02; for 10 mg/kg, *p <* 0.01) significantly restored the lower sucrose intake in CUMS-treated animals.

### 3.3. Isofraxidin Increases CUMS-Induced Decrease of Locomotor Activity in OFT

**Figure 4:**
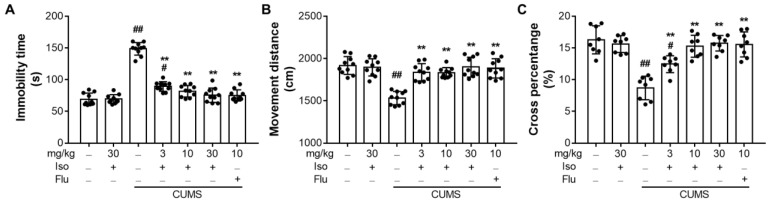
Isofraxidin increases CUMS-induced decrease of locomotor activity in OFT. (**A**) Total immobility time. (**B**) Total movement distances. (**C**) Percentage of time for mice reaching the central area. Data are presented as means ± SEM (*n* = 10). The Control group was CUMS-free mice administered with normal saline. CUMS group was CUMS mice. Significance was analyzed by one-way ANOVA followed by the Bonferroni correction. # *p <* 0.05 and ## *p <* 0.01, compared with the control group. ** *p <* 0.01, compared with CUMS group.

As shown in Figure 4, the CUMS elevated the immobility duration (Figure 4A; F (6, 63) = 94.72; *p* < 0.01), but decreased total movement distances (Figure 4B; F (6, 63) = 18.55; *p <* 0.01) and the percentage of time passing the center (Figure 4C; F (6, 49) = 20.72; *p <* 0.01) in mice compared to the control group. However, treatments with isofraxidin (F (6, 63) = 18.55; for 3, 10, and 30 mg/kg, all *p <* 0.01) or fluoxetine hydrochloride (F (6, 63) = 18.55; for 10 mg/kg, *p <* 0.01) restored these inclinations dramatically. In addition, fluoxetine might exacerbate the reduction in locomotor activity generated by CUMS in OFT.

### 3.4. Isofraxidin Alleviates CUMS-Induced Increase of Immobility Duration in FST and TST

**Figure 5:**
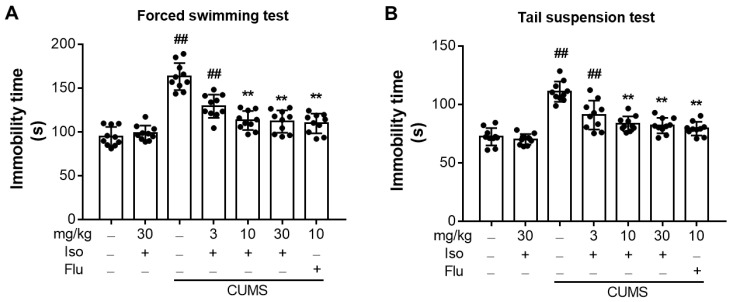
Isofraxidin alleviates CUMS-induced increase of immobility duration in FST and TST. (**A**) Duration of immobility in FST. (**B**) Total immobility time in TST. Data are presented as means ± SEM (*n* = 10). The Control group was CUMS-free mice administered with normal saline. CUMS group was CUMS mice. Significance was analyzed by one-way ANOVA followed by the Bonferroni correction. ## *p <* 0.01, compared with the control group. ** *p <* 0.01, compared with CUMS group.

The duration of immobility in FST (Figure 5A; F (6, 63) = 36.96, *p <* 0.01) and TST (Figure 5B; F (6, 63) = 31.23, *p <* 0.01) was significantly increased in the CUMS-stimulated group from that of the control group. But, isofraxidin (10 and 30 mg/kg) treatments could significantly alleviate immobility durations in both FST (F (6, 63) = 36.96; for 3, 10, and 30 mg/kg, all *p <* 0.01) and TST (F (6, 63) = 31.23; for 3, 10, and 30 mg/kg, all *p <* 0.01), compared with the CUMS group. Also, fluoxetine (10 mg/kg) reduced the duration of immobility caused by CUMS.

In conclusion, these behavioral tests demonstrated that 10 and 30 mg/kg of isofraxidin exerted almost the same inhibitory effects on CUMS-induced depressive behaviors. Thus, the dose of 10 mg/kg was then selected and used for our further experiments and mechanistic investigations.

### 3.5. Isofraxidin Attenuates CUMS-Induced Hyperactivity of the Hypothalamic-Pituitary-Adrenal Axis

**Figure 6:**
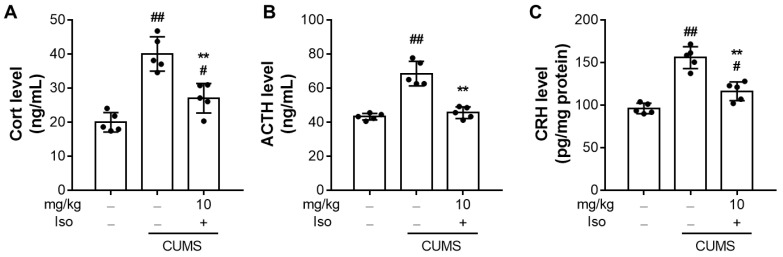
Isofraxidin attenuates CUMS-induced hyperactivity of the hypothalamic-pituitary-adrenal axis. (**A**–**C**) Levels of serum Cort (**A**) and ACTH (**B**) and hypothalamus CRH (**C**) in mice. The neuroendocrine hormones were determined by ELISA. The Cort and ACTH levels were normalized to the serum volume and the CRH level was normalized to the total protein. Data are presented as means ± SEM (*n* = 5). The Control group was CUMS-free mice administered with normal saline. CUMS group was CUMS mice. Significance was analyzed by one-way ANOVA followed by the Bonferroni correction. # *p <* 0.05 and ## *p <* 0.01, compared with the control group. ** *p <* 0.01, compared with CUMS group.

As shown in Figure 6A,B, CORT (F (2, 12) = 29.76; *p <* 0.01) and ATCH (F (2, 12) = 43.88; *p <* 0.01) significantly increased in the CUMS group compared to the control group. Nevertheless, treatments with isofraxidin (10 mg/kg) significantly decreased CORT (F (2, 12) = 29.76; *p <* 0.01) and ATCH (F (2, 12) = 43.88; *p <* 0.01) levels, compared to the CUMS group. In addition, isofraxidin (10 mg/kg) decreased the levels of CRH in the hypothalamus (Figure 6C; F (2, 12) = 42.62; *p <* 0.01), compared with CUMS group.

### 3.6. Isofraxidin Suppresses CUMS-Induced Increased Expression of Pro-Inflammatory Cytokines

**Figure 7:**
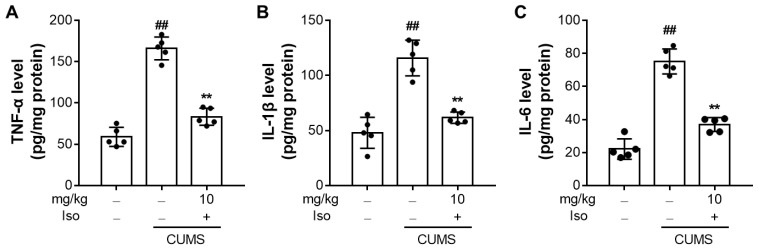
Isofraxidin suppresses CUMS-induced increased expression of pro-inflammatory cytokines. Levels of TNF-α (**A**), IL-1β (**B**), and IL-6 (**C**) in the hippocampus were determined by ELISA and normalized to protein levels. Data are presented as means ± SEM (*n* = 5). The Control group was CUMS-free mice administered with normal saline. CUMS group was CUMS mice. Significance was analyzed by one-way ANOVA followed by the Bonferroni correction. ## *p <* 0.01, compared with the control group. ** *p <* 0.01, compared with CUMS group.

Compared to the control group, CUMS substantially elevated the protein expressions of TNF-α (F (2, 12) = 109.8; *p <* 0.01), IL-6 (F (2, 12) = 98.85; *p <* 0.01), and IL-1β (F (2, 12) = 39.68; *p <* 0.01) in the hippocampus (Figure 7). Notably, isofraxidin (10 mg/kg) significantly attenuated CUMS-induced elevated expressions of TNF-α (F (2, 12) = 109.8; *p <* 0.01), IL-6 (F (2, 12) = 98.85; *p <* 0.01), and IL-1β (F (2, 12) = 39.68; *p <* 0.01), compared with the CUMS group.

### 3.7. Isofraxidin Inhibits CUMS-Induced Activation of NF-κB and NLRP3 Inflammasome Pathways

**Figure 8:**
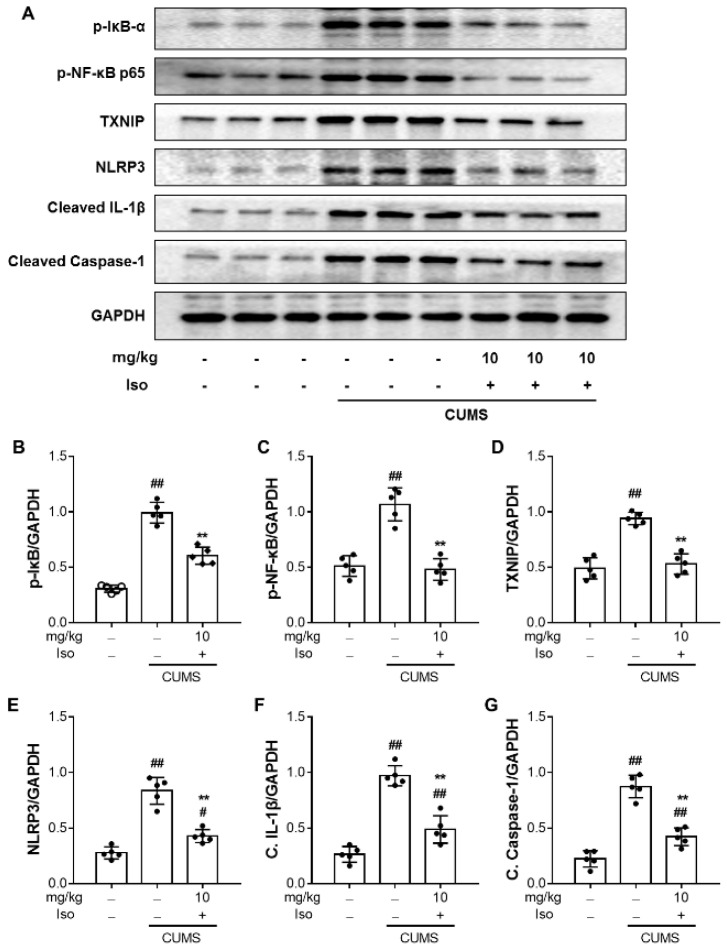
Isofraxidin inhibits CUMS-induced activation of NF-κB and NLRP3 inflammasome pathways. Level of phosphorylated IκB α (**A**,**B**), phosphorylated p-NF-κB p65 (**A**,**C**), TXNIP (**A**,**D**), NLRP3 (**A**,**E**), cleaved IL-1β (**A**,**F**), and cleaved caspase-1 (**A**,**G**) in different brain samples. The protein level was normalized to and expressed as folds of GAPDH. Data are presented as means ± SEM (*n* = 5). The Control group was CUMS-free mice administered with normal saline. CUMS group was CUMS mice. Significance was analyzed by one-way ANOVA followed by the Bonferroni correction. # *p <* 0.05 and ## *p <* 0.01, compared with the control group. ** *p <* 0.01, compared with CUMS group.

To investigate the impact of isofraxidin on the NF-κB axis, p65 levels in the hippocampus were measured. As illustrated in Figure 8, CUMS stimulation resulted in substantial phosphorylation of NF-κB p65 (F (2, 12) = 40.58; *p <* 0.01) in the hippocampus, compared with the control group. Nonetheless, isofraxidin (10 mg/kg) treatment effectively inhibited NF-κB phosphorylation (Figure 8A,C; F (2, 12) = 40.58; *p <* 0.01). Moreover, as shown in Figure 8A,B, we discovered that CUMS significantly elevated the amount of phosphorylated IκB (F (2, 12) = 113.5; *p <* 0.01), which was reversed by additional isofraxidin (10 mg/kg) treatment (F (2, 12) = 113.5; *p <* 0.01).

Components of the inflammasome complex were also determined to explore whether the NLRP3 pathway was implicated in the effects of isofraxidin. As shown in Figure 8, CUMS significantly enhanced the expression of TXNIP (Figure 8A,D; F (2, 12) = 45.01; *p <* 0.01) and NLRP3 inflammasomes (Figure 8A,E; F (2, 12) = 60.31; *p <* 0.01) in the hippocampus, which were reversed by isofraxidin (10 mg/kg). In addition, the maturation of IL-1β (Figure 8A,F; F (2, 12) = 68.89; *p <* 0.01) and caspase-1 (Figure 8A,G; F (2, 12) = 75.58; *p <* 0.01) were significantly elevated in the hippocampus following CUMS, which were reversed by isofraxidin (10 mg/kg).

## 4. Discussion

CUMS is commonly utilized to simulate the etiology and behavior of depression in animals [30,31,32,33]. In the present investigation, stimulation with CUMS was able to induce a depressed phenotype in mice, as demonstrated by a substantial decrease in sucrose consumption and the disappearance of anhedonia. Then, we discovered that isofraxidin treatment could restore the impaired sucrose preference in CUMS-treated mice, indicating that indeed isofraxidin had antidepressant-like actions. The OFT is commonly used to examine locomotor activity and exploratory behaviors, whereas the FST and TST are used to assess immobility, the distinctive behavior index of behavioral despair observed in humans [18,19]. We demonstrated the antidepressant-like effects of isofraxidin in mice with CUMS-induced depression, based on the fact that isofraxidin improved locomotor activity in the OFT and decreased immobility lengths in the FST and TST. Thus, these data suggested that isofraxidin had potential antidepressant properties.

According to previous research, stress-induced depressive behavior is associated with the activation of the HPA axis. Diverse types of powerful stressors can cause HPA axis hyperactivity, resulting in blood CORT and ACTH release and hypothalamic CRH expression [34,35]. Treatment with several well-known antidepressants may reduce the stress-induced rise in blood CORT and hypothalamus CRH expression [34,35]. Following earlier investigations, CUMS-induced animals exhibited activation of the HPA axis. Moreover, isofraxidin dramatically reduced the blood corticosterone level and CRH expression in the hypothalamus of mice with CUMS. Consequently, our findings revealed that the antidepressant-like effectiveness of isofraxidin was accompanied by a regulating impact on the hyperactivity of the HPA axis.

A growing body of research shows that CNS inflammation and depression go hand in hand and that the pathophysiology of depressive illnesses is significantly influenced by the overproduction of inflammatory cytokines and mediators [9,10,11,12,13,14,15,16]. In the CNS, pro-inflammatory cytokines typically exist in low concentrations and regulate neural function and neuronal survival [9,10]. However, pro-inflammatory cytokines that are significantly produced in the brain in depressed models brought on by CUMS limit the release of neurotrophic factors and the repair of neurons. Therefore, it has been demonstrated that cytokines such as TNF-α, IL-1β, and IL-6 are important mediators of depressed-like behavior and may even be utilized to predict the onset of depressive symptoms [36]. In this investigation, we showed that isofraxidin inhibits the production of TNF-α, IL-1β, and IL-6 that are elevated by CUMS. Additionally, other antidepressants including paroxetine and sertraline have been demonstrated to have an inhibitory impact on the generation of inflammatory cytokines [37,38,39]. Furthermore, IL-1 family production suppression gives anti-depressive effects, such as a reduction in anhedonia and locomotor activity, whereas TNF-α overbalances in the brain also result in the development of depressive-like behavior [37,38,39]. Overexpression of TNF-α, IL-6, and IL-1β is also known to cause the HPA axis to become hyperactive and to lower levels of neurotrophic factors [40,41]. The current investigation indirectly supported the inhibitory impact of isofraxidin on CUMS-induced neuroinflammatory responses by confirming the isofraxidin-mediated inhibition on hyperactivity of the HPA axis and enhancement of neurotrophic factors. Accordingly, the present findings showed that the antidepressant-like effects of isofraxidin were also supported by its inhibitory responsibilities on the production of inflammatory cytokines and mediators in the brain of CUMS animals.

The regulation of pro-inflammatory cytokines is a well-known function of NF-κB and NLRP3 inflammasomes [11,12,13,14,15,16,17,18,19]. Inactive NF-κB often binds to the IκB proteins and accumulates in the cytoplasm [11,12,13,14,15,16,17,18,19]. The IκB kinase first phosphorylates and degrades IκB, which causes NF-κB to become dissociated from its complex and stimulates NF-κB translocation into the nucleus [11,12,13,14,15,16,17,18,19]. In CUMS-induced depressed models, the NF-κB pathway activation has been extensively documented, and NF-κB inactivation limits the inflammatory response and recovers depressive-like behaviors. In prior investigations, the inhibitory impact of isofraxidin on NF-κB activation was demonstrated in sepsis and acute lung damage caused by lipopolysaccharide [22,23,24,25,26]. Additionally, in the current investigation, we first showed that isofraxidin might limit NF-κB activation by reducing NF-κB phosphorylation and suppressing IκB kinase phosphorylation and degradation. These results partially confirmed our hypothesis that isofraxidin inhibited pro-inflammatory cytokines by controlling the NF-κB pathway. Another frequent regulator of inflammatory processes is the activation of the NLRP3 inflammasome [40,41]. The NLRP3 inflammasome facilitates the conversion of precursor caspase-1 and IL-1β into cleaved caspase-1 and mature IL-1β, respectively, by interacting with an adapter protein called ASC. The extracellular environment was then exposed to active caspase-1 and mature IL-1β, which support inflammatory and apoptotic processes [40,41]. According to recent research, NLRP3 inflammasome activation in the hippocampus of mice treated with CUMS as well as its downstream targets (ASC, cleaved caspase-1, and mature IL-1β) could be suppressed by isofraxidin. We hypothesized that the isofraxidin inhibitory effect on NF-κB transactivation may play a role in the inactivation of the NLRP3 inflammasome given that it has been documented that the NF-κB signals transduction is what drives NLRP3 inflammasome production. As a result, isofraxidin (10 mg/kg) was able to reverse CUMS-induced changes in pro-inflammatory cytokines via NF-κB and NLRP3 signaling pathways, which may explain some of its antidepressant effects.

## 5. Conclusions

The main conclusions of this study were as follows: (1) Isofraxidin was able to alleviate depressive-like behaviors in CUMS-induced mice model; (2) The effects were accompanied by its inhibitory roles on hyperactivity of the HPA axis and expression of pro-inflammatory cytokines; and (3) Isofraxidin-mediated antidepressant-like effects were associated with NF-κB /NLRP3 inflammasome pathways.

## Data Availability

The data presented in this study are available in the article.
